# Development of improved vaccine cell lines against rotavirus

**DOI:** 10.1038/sdata.2017.21

**Published:** 2017-03-01

**Authors:** Weilin Wu, Nichole Orr-Burks, Jon Karpilow, Ralph A. Tripp

**Affiliations:** 1 Department of Infectious Diseases, College of Veterinary Medicine, University of Georgia, Athens, Georgia 30602, USA; 2 Proventus Bio, 220 Riverbend Rd, Athens, Georgia 30602, USA

**Keywords:** RNAi, Rotavirus, Vaccines, High-throughput screening

## Abstract

Rotavirus is a major cause of severe gastroenteritis among very young children. In developing countries, rotavirus is the major cause of mortality in children under five years old, causing up to 20% of all childhood deaths in countries with high diarrheal disease burden, with more than 90% of these deaths occurring in Africa and Asia. Rotavirus vaccination mimics the first infection without causing illness, thus inducing strong and broad heterotypic immunity against prospective rotavirus infections. Two live vaccines are available, Rotarix and RotaTeq, but vaccination efforts are hampered by high production costs. Here, we present a dataset containing a genome-wide RNA interference (RNAi) screen that identified silencing events that enhanced rotavirus replication. Evaluated against several rotavirus vaccine strains, hits were validated in a Vero vaccine cell line as well as CRISPR/Cas9 generated cells permanently and stably lacking the genes that affect RV replication. Knockout cells were dramatically more permissive to RV replication and permitted an increase in rotavirus replication. These data show a means to improve manufacturing of rotavirus vaccine.

## Background & Summary

Rotaviruses (RV) belong to the *Reoviridae* family, and have a linear segmented double-stranded RNA genome composed of 11 segments coding for 12 viral proteins. RV is a major cause of severe gastroenteritis among very young children in developing countries, and is a major cause of mortality in children under 5 years old, causing up to 20% of all childhood deaths in countries with high diarrheal disease burden with >90% of these deaths occurring in Africa and Asia. Initial RV infections usually occur by 9 months of age, and 80% occur before 1-year of age^1–5^. Serogroup A RV are the leading causative agent of childhood (<5 years) severe gastroenteritis worldwide, accounting for approximately 3.4% of child deaths annually^6^. The WHO has recommended, since 2009, that rotavirus vaccines be included in all national vaccination programs^7^.

There are two RV vaccines in use. Rotarix is a live vaccine containing the attenuated monovalent G1, P^8^ human rotavirus strain. RotaTeq is a live-attenuated bovine-human reassortant rotavirus vaccine containing the most common rotavirus antigens seen in humans (G1, G2, G3, G4, and P). Both vaccines are efficacious as has been demonstrated in clinical trials, i.e., 90–100% effective in preventing severe gastroenteritis, and clinical trial data have shown both vaccines to have acceptable safety profiles. Both vaccines are orally administered in multiple doses to mimic natural sequential rotavirus infections in an effort to promote the development of homotypic and heterotypic protective immunity against relevant group A rotavirus^8^. Current efforts to disseminate these vaccines to economically distressed areas are hindered largely by bioproduction costs.

**Figure 1:**
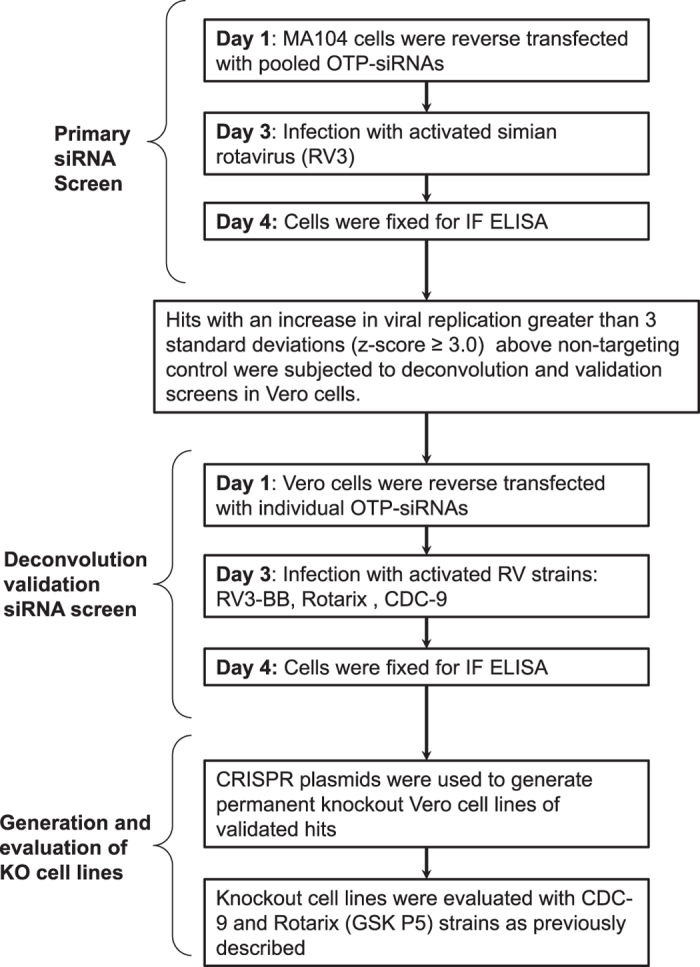
Experimental workflow. This study included a tiered siRNA screening approach coupled with CRISPR-generated knockout Vero cell lines to evaluate host genes and their implication during RV infection. The primary siRNA screen was performed with pooled OTP-siRNA in MA104 cell line using the simian rotavirus strain RV3. Top hits were selected by a z-score analysis and subjected to deconvoluted validation siRNA screens in Vero cells with the rotavirus strains RV3-BB, Rotarix, CDC-9. Resulting hits were evaluated in CRISPR generated Vero cell lines with CDC-9 and Rotarix (GSK P5) strains.

**Figure 2:**
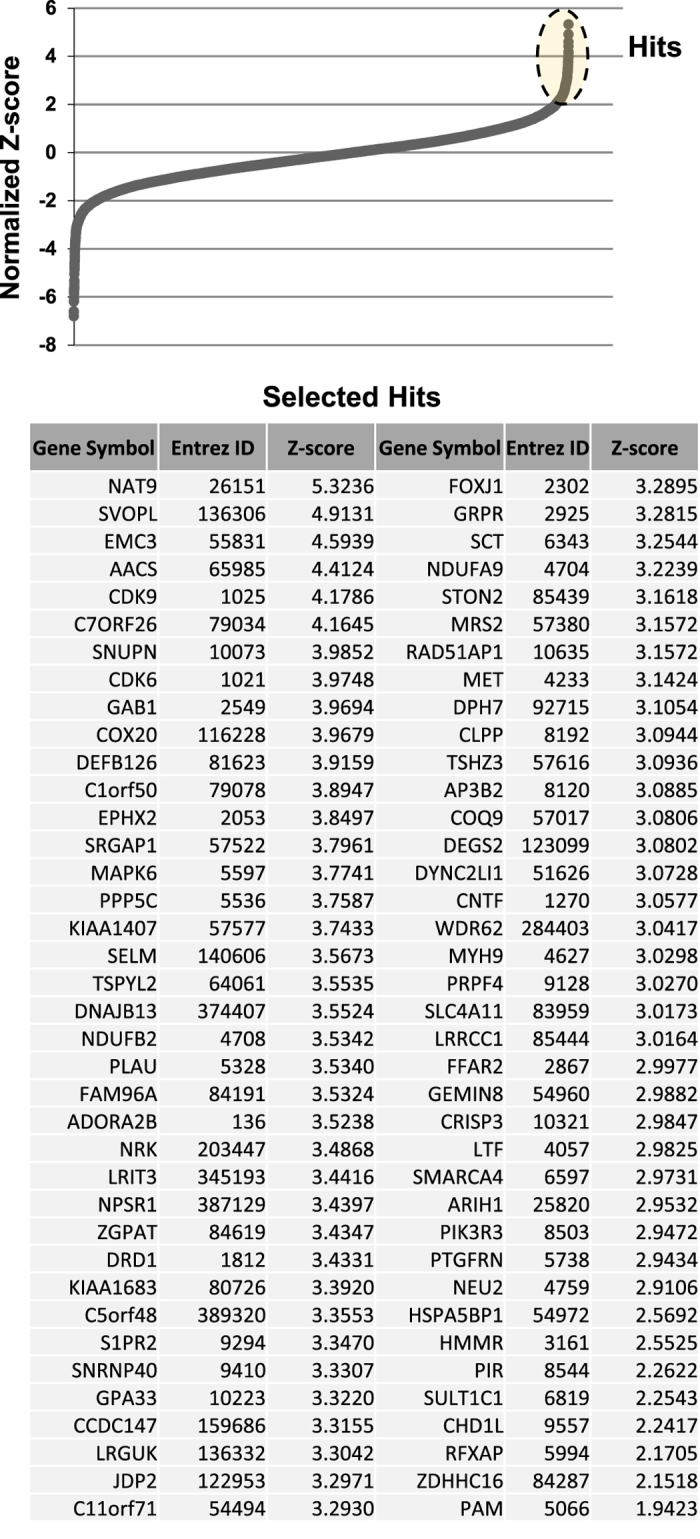
Primary genome-wide siRNA screen in MA104 cell line. Eighteen thousand two hundred forty genes were targeted with siRNAs. Forty-eight hours post-transfection cells were infected with the strain RV3. Twenty-four hours post-infection the cells were fixed and IF ELISA was completed. KD of 76 genes resulted in an enhancement of viral replication ≥3 s.d.s. (z-score≥3.0) as compared to non-targeting control. All samples were completed in triplicate (*n*=3). These 76 genes were validated by deconvolution of pooled siRNAs.

**Figure 3:**
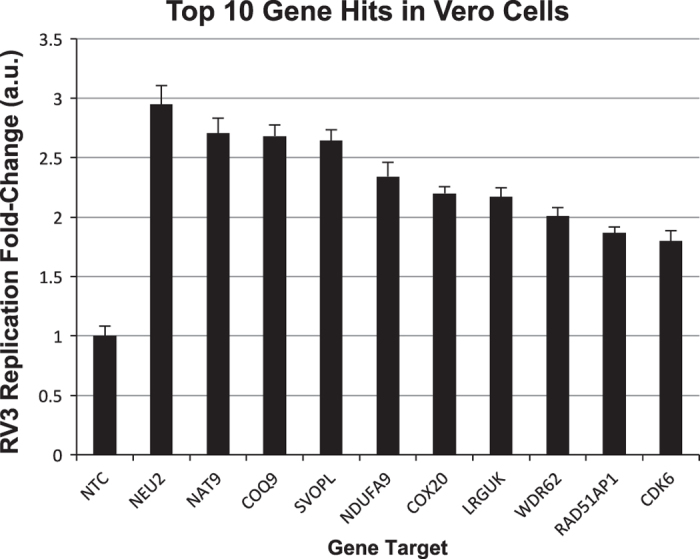
Deconvolution and validation of hits in Vero cell line. Presented is the relative fold-change of RV3 replication in siRNA transfected Vero cells compared to non-targeting control cells. The data is shown as mean values±s.d. To efficiently remove false positive from the siRNA primary screen in MA104, we re-screened these 76 hits using SMARTpool siRNAs targeting these genes in Vero cells. siRNAs were transfected into Vero cells following the methods optimized for Vero cells. Forty-eight hours post-siRNA transfection, cells were infected with RV3 using an MOI=0.2, and cells were harvested for RV3 IF ELISA 48 h.p.i. All samples were completed in triplicate (*n*=3). a.u., arbitrary units.

**Figure 4:**
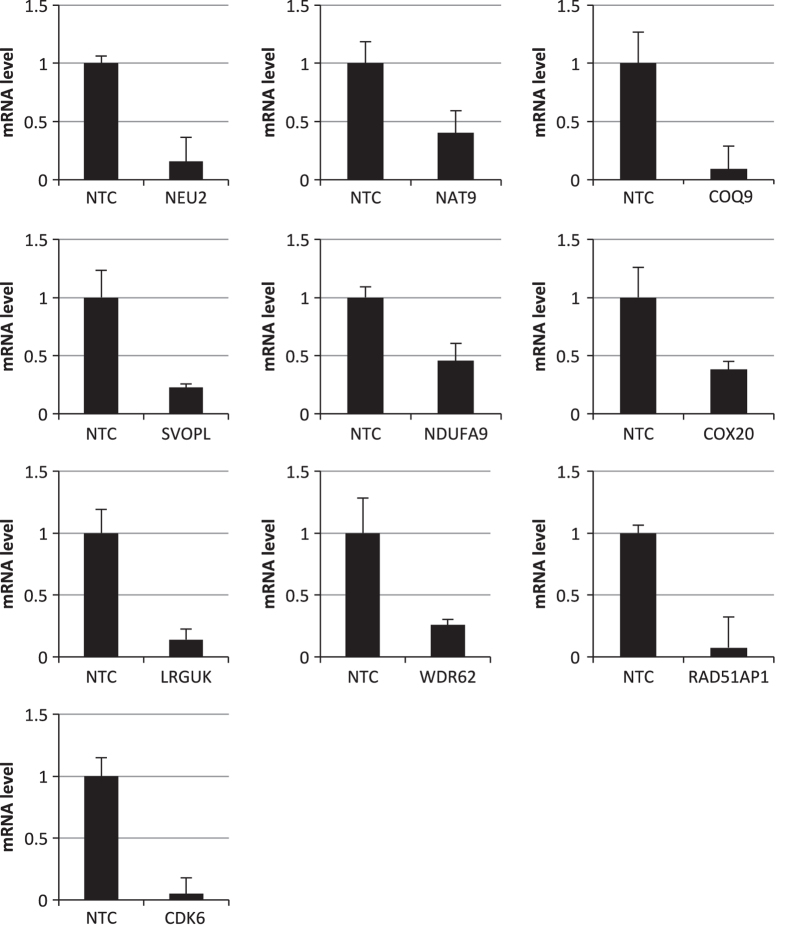
Validation of gene silencing by siRNA transfection in Vero cell line using qPCR. For the top 10 hits selected to perform gene editing the siRNA silencing was confirmed in Vero cells using SMART pool siRNAs. MRNA levels of each gene were measured 48 h post-siRNA transfection by qPCR. Each qPCR reaction utilized 1 ug RNA as part of the reverse transcription reaction. Each gene was evaluated in triplicate and average values normalized to 18s rRNA. Values are presented as fold-change compared to non-targeting control (NTC). All samples were completed in triplicate (*n*=3). Values represent the mean of the triplicates±s.e.m.

**Figure 5:**
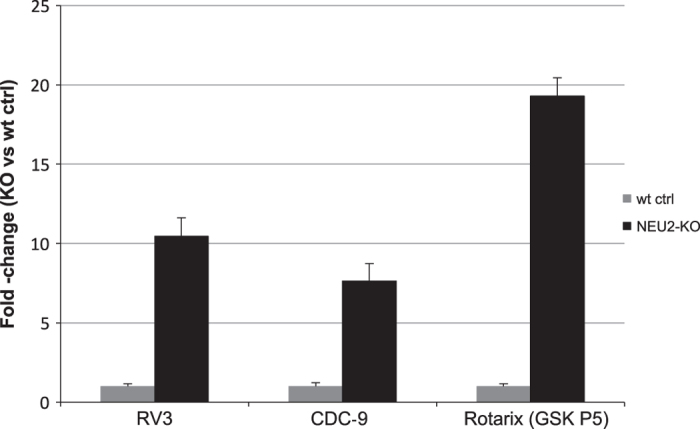
RV replication in NEU2-KO Vero cells. Using the CRISPR-Cas system a functinal NUE2 gene was edited and the RV3, CDC-9 and Rotarix rotavirus strains were evaluated. QPCR was performed in triplicate and the value was normalized to 18s RNA and compared to wild type Vero cell control (wt ctrl). All samples were completed in triplicate (*n*=3). The value presented here represents the mean of the triplicates±s.e.m.

Mammalian cells such as the Vero cell line have proven to be a safe production platform for developing human vaccines^7,9^. Unfortunately, currently available Vero vaccine cell lines have relatively low yields compared to other vaccine production platforms. In an effort to rectify low-yield issues, as part of this study, we have identified host virus-resistant genes in a monkey kidney cell line, MA104 cells. The MA104 cells are highly susceptible to RV. Using small interfering RNA (siRNA) human library screening, a subset of genes in MA104 cells were identified that considerably increased RV replication when knocked down. The primary siRNA screen in the MA104 cells was achieved by reverse-transfecting the cells with pooled ON-TARGETplus siRNAs (SMARTpools) (4 siRNA targeting each gene) with the siRNA library targeting protein-coding host genes. Cytotoxic siRNAs as well as those which negatively affected cell viability were identified and excluded. Two days post-transfection cells were infected with activated simian rotavirus (RV3). Twenty-four hours post-infection cells were fixed and virus quantified by fluorescence focus assay (IF ELISA) (Fig. 1). Seventy hits increased viral replication ≥3 s.d.s. (z-score≥3.0) above non-targeting control (Fig. 2). A WHO-derived Vero cell line, previously utilized in the production of vaccine cell lines, was used to re-screen hits in an effort to eliminate false-positives. The top 20 hits that recapitulated the primary MA104 cell line screen were subjected to deconvolution studies. In this case, individual siRNAs included in SMARTpools were tested individually to determine if they induced the phenotype observed of the pool, i.e., increased RV replication and changes in viral antigen production. As part of this validation screen, Vero cells were reverse-transfected with individual siRNAs (4 siRNAs per gene) in triplicate per a transfection protocol optimized for Vero cells. Forty-eight hours post-transfection cells were infected with the RV3 rotavirus strain. Viral replication was evaluated via IF ELISA (Fig. 3). Ten hits showed an increased in RV3 replication of 1.75 fold or higher (NEU2, NAT9, COQ9, SVOPL, NDUFA9, COX9, LRGUK, WDR62, RAD51AP1 and CDK6) (Table 1). Messenger RNA knockdown (KD) under infection conditions was confirmed with qRT-PCR (Fig. 4). Gene knockout (KO) Vero cell lines were generated with CRISPR-cas 9 plasmids. KO was confirmed via Sanger sequencing (data not shown). RV3, CDC-9 and Rotarix replication was evaluated by qPCR in these engineered cell lines. In the NEU2-KO Vero cells generated, Rotarix infection had the highest increase of viral transcript at ~18-fold increase, followed by the RV3 (reference strain) at ~10-fold, and CDC-9 at ~7.5-fold increase (Fig. 5).

## Methods

### Cell culture and viral stocks

The rhesus monkey epithelial cell line (MA104) was chosen to perform the primary RNAi screen because these cells support rotavirus replication and are easily transfected. The effects of gene silencing on rotavirus replication in MA104 cells can be detected 16–24 h (h) post-infection (pi). Hits produced from this primary screen were validated in the African green monkey kidney cell line (Vero), which is used for the production of the licensed polio virus vaccine. Vero cells are an approved cell line for vaccine production, and the cell line was received from the Centers of Disease Control and Prevention (CDC, Atlanta) who obtained it from the Serum Institute of India (SI). Both cell lines were cultivated under conditions of 37 °C, 5% CO_2_ in Dulbecco’s modified Eagle’s medium (DMEM; Hyclone, GE Healthcare) supplemented with 10% heat inactivated fetal bovine serum (FBS; Hyclone, GE Healthcare) and 1% penicillin-streptomycin. A master cell stock was created with low passage cells for both cell types. Briefly, master cell stocks were generated for low-passaged Vero and MA104 cells, where cells were cultured 6 days prior to screening experiments. The primary RNAi screens were done in MA104 cells by seeding MA104 cells in 96-well plates at a cell density of 10,000 cells per well. Rescreening and validation screening was accomplished in the same 96-well plate format with Vero cells seeded at a cell density of 8,000 cells per well. Passage numbers were consistent throughout the screen and validation steps to reduce variability.

The primary RNAi screening was done using a single lot of a attenuated RV vaccine strain, RV3-BB (RV3)^10^. RV3 was initially prepared as a vaccine at a low titer (6.5×10^5^ FFU ml^−1^) in Melbourne, Australia by the Carl Kirkwood lab. High titer RV3 virus was produced in the WHO-approved Vero cell line under Good Manufacturing Practice (GMP) conditions at Meridian Life Sciences, Memphis. RV3 is currently propagated in monkey kidney cell cultures (Vero cells) and subjected to downstream purifications. We expect that other RV strains may share the same pathways for virus replication as the currently licensed RV3 vaccine strain. For this reason, other RV strains were examined as part of the validation experiments to determine whether silencing the top hits determined as part of the primary screening with RV3 also lead to increased viral replication in vaccine cell lines with a variety of RV strains. Subsequent validation steps were completed in Vero cells with the RV3-BB strain, RotaTeq (G1P7, G2P7, G3P7, G4P7, G6P1A), Rotarix (89-12/G1P) and CDC-9 rotavirus strains.

### SiRNA transfection

An ON-TARGETplus small interfering RNA (SMARTpools) library (GE Healthcare) was utilized to screen protein-coding host genes in MA104 cells as part of the primary screen. Primate-specific siRNA libraries are not currently readily available, therefore we used a human library of siRNAs to target the entire genome. According to the Chimpanzee Sequencing and Analysis Consortium, primates share 96% DNA sequence identity with humans. Therefore, utilizing siRNAs targeting human genes should target monkey sequences with strong complementarity^11^. SMARTpools utilize sense strand inaction and seed-region modifications that reduce off-target effects while maintaining high selectivity and effectiveness. As part of the primary RNAi screen, each gene was targeted with pooled SMARTpools. Each pool contained four siRNAs targeting a different seed region of the target gene’s mRNA. Briefly, in a 96-well plate format, MA104 cells were reverse transfected with 50 nM siRNA pools using 0.4% DharmaFECT 4 transfection reagent (0.4 ul well^−1^) (GE Healthcare). SMARTpools were pre-incubated with DharmaFECT 4 reagent in serum-free DMEM media at room temperature for 20 min to allow for complex formation. MA104 cells (1.4×10^4^ cells well^−1^) in DMEM supplemented with 10% heat inactivated fetal bovine serum (FBS) were added to each well. Transfection plates were cultured at 37 °C, 5% CO_2_. Forty-eight hours post-transfection media was removed and cells infected with rotavirus strain as previously stated. Subsequent deconvolution studies utilized the same method of transfection except each siRNA was delivered individually. Each transfection experiment includes a non-targeting control siRNA (NTC; GE Healthcare). This siRNA targets no known mRNA sequence and should have no effect on mRNA. A siRNA targeting RV3 was used as a positive control, siTOX (GE Healthcare) served as a transfection control. All siRNAs were transfected to a final concentration of 50 nM.

### Elimination of false-positive hits

Genome-wide RNAi screen data shows that the percentage of false-positive hits produced following primary screening can be high, in part due to the working with high numbers of assays including transfection, infection, fixation, variations from plate to plate, and delays in sequential plate reads. In the current study, the primary siRNA screening was performed in a MA104 cell line, which supports rotavirus replication. However, our endpoint for gene editing was performed in a Vero cell line that we used to support current vaccine development. To help aid in removal of potential false-positive hits in the primary screening, we performed a secondary screen of this collection of hits in a Vero cell line. The pooled siRNAs were transfected into Vero cells in a 96-well cell culture plate in triplicate. Rotavirus replication was examined by ELISA assay and the data was normalized to non-targeting control (NTC) as described. Seventy-six genes were selected for secondary screening in the Vero cell line. The majority of genes selected for re-screening studies were selected based upon z-score analysis. Initially, genes that exhibited a z-score≥3.0 were included. This list was further modified to include genes that were predicted to be relevant during virus replication following a brief study that assessed genes with a z-score ranging from 2.9–3.0 by conducting basic pathway analysis and a literature search. Genes were excluded during this process based upon their predicted relevance to virus replication. The finalized list of 76 genes is outlined in Fig. 2.

### Deconvolution studies

Twenty hits that repeated in the primary screen results following screening in Vero cells were chosen based upon the increased virus replication with a relative value >1.4 (normalized to NTC) and subjected to deconvolution studies (Table 2 (available online only)). As part of these studies the siRNAs included in each SMARTpool were individually tested to determine whether individual siRNAs gave rise to changes in viral antigen production determined by IF ELISA. For each gene, 4 individual siRNAs from SMARTpools were evaluated. Forty-eight hours post-transfection, the cells were infected with RV3 at a MOI 0.2. Forty-eight hours post-infection, cells were fixed for the IF ELISA assay as discussed in methods section. A siRNA targeting RV3 was used as a positive control, siTOX served as a transfection control and a non-target siRNA served as a negative control. Gene targets were considered confirmed if two individual siRNAs enhanced RV3 replication during the deconvolution screening with a relative value >1.4 (normalized to NTC). To confirm siRNA efficacy, mRNA levels for each gene target were evaluated following transfection and subsequent infection by qPCR. All data represents the mean of triplicates±s.e.m., and normalized to non-targeting control.

### Toxicity and cellular viability screening

**Figure 6:**
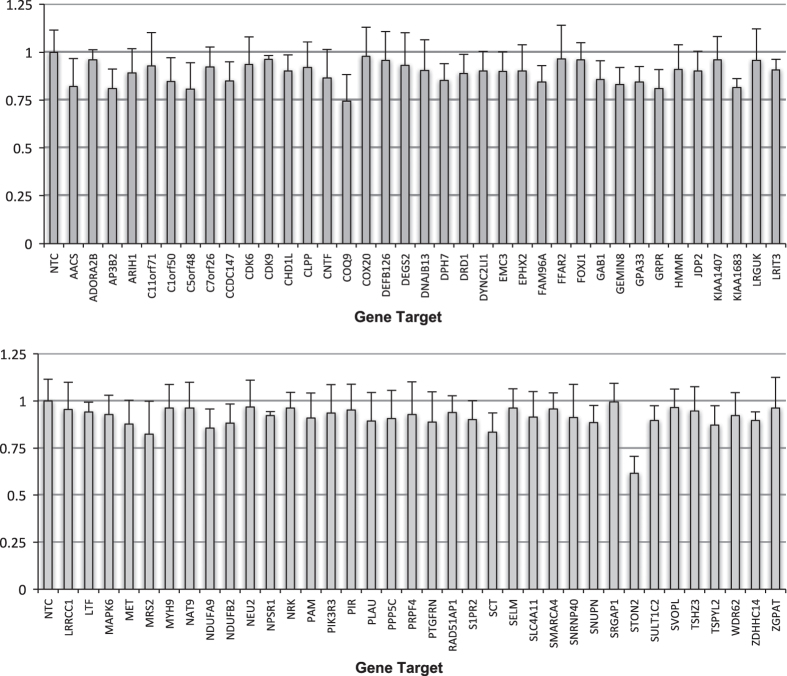
Cell viability assay. siRNAs were reverse-transfected into MA104 cells (10,000 per well) to a final concentration of 50 nM. Seventy-two hours post-transfection, 20 μl per well of CellTiter 96 Non-Radioactive Cell Proliferation Assay (G4000, Promega Inc) was added to the siRNA transfected cells and incubated at 37 °C in cell culture incubator for 1 h. Following incubation, 100 μl of the Solubilization Solution/Stop Mix was added to each well. Absorbance was evaluated at a wavelength of 570 nm with a plate spectrophotometer (Tecan).

The top 76 hits were tested for effects on cellular viability and toxicity by CellTiter96 Non-Radioactive Cell Proliferation Assay Kit and ToxiLight bioassay (Lonza, Walkersville, MD) (Fig. 6). SiRNAs were reverse transfected into MA104 cells (10,000 per well) to a final concentration of 50 nM. Seventy-two hours post-transfection, 20 μl per well of CellTiter 96 Non-Radioactive Cell Proliferation Assay (G4000, Promega Inc) was added to the siRNA transfected cells and incubated at 37 °C in cell culture incubator for 1 h. Following incubation, 100 μl of the solubilization solution/stop mix was added to each well. Absorbance was evaluated at a wavelength of 570 nm with a plate spectrophotometer (Tecan). Absorbance values were normalized to non-targeting siRNA control absorbance values.

### qRT-PCR

Gene silencing by siRNA transfection in Vero cells was confirmed for the top ten genes. Cells were transfected in triplicate with individual siRNAs as previously described. Forty-eight hours post-transfection, cells were harvested for RNA isolation using RNeasy Mini Kit (Qiagen) following manufacturer instructions. qRT-PCR was performed in triplicate to measure mRNA levels of targeted genes using 1 ug RNA (SYBR Green RT Kit; ThermoFisher). The cycle threshold (CT) values were normalized to the house keeping gene glyceraldehyde 3-phosphate dehydrogenase (GAPDH) or 18s ribosomal RNA. Values are presented as the mean of the triplicates±s.e.m.

### RV infection

For both primary and deconvolution experiments, MA104 cells or Vero cells were infected with an activated simian rotavirus (RV3) 48 h post-transfection. Briefly, RV stocks were activated by adding 10 ug ml^−1^ of L-(tosylamido-2-phenyl) ethyl chloromethyl ketone (TPCK) treated trypsin (Worthington; Lakewood, NJ) and heating for 1 h in a 37 °C water bath. Following activation, rotavirus was diluted to a multiplicity of infection (MOI) of 0.2 in media containing trypsin at a 1 ug ml^−1^ concentration. One hundred microliters of diluted RV was added to each well after first washing plates ×2 with HBSS to remove all traces of FBS. Infected plates were incubated for 4 h at 37 °C, 5% CO_2_. Subsequent to incubation, 100 ul of DMEM containing 10% FBS was added to the plates on top of existing infection and incubated for a total of 24 h under the same conditions. Twenty-four hours post-infection supernatants were harvested and cells fixed with acetone:PBS for 20–30 min and stored at 4 °C until ready to perform the IF ELISA. KO cell lines were examined for virus replication using two RV vaccine strains CDC-9 and Rotarix (GSK P5) (CDC) following the same protocol.

### IF ELISA

Fixed plates were washed ×2 with PBS for rehydration then blocked with 0.05% phosphate buffered saline with Tween 20 (PBST) containing 1% BSA for 1 h at room temperature on a rotator. The primary polyclonal rabbit anti-Rotavirus antibody (Rab A-SA11; Melbourne, Australia) 1:1,000 in blocking solution was added at 50 ul well^−1^ for 1 h at room temperature. The primary antibody was removed and plates were washed ×4 with 0.05% PBST followed by the addition 50 ul well^−1^ of a fluorescently labeled secondary antibody (goat/Anti-rabbit Alexa 488) at 1:1,000 dilution for 1 h at room temperature. Secondary antibody was removed by washing ×2 with PBST and ×2 with PBS. Plates were read with the Beckman Coulter Paradigm spectrophotometer capable of detecting fluorescence. Plates were read individually using a 450 nm wavelength filter at an exposure time of 1 min. Values were normalized to non-targeting control wells in the same plate.

### CRISPR-Cas9

Following the re-screening and deconvolution studies, KOs of the top 10 hits were generated in Vero cells using CRISPR-Cas9. These 10 genes were chosen based upon assay results i.e., they resulted in the highest fold-change compared to NTC (enhanced virus replication versus non-targeting control). CRISPRs (GE) were used to generate 5 of the 10 knockouts. Utilization of the CRISPRs requires the co-transfection of a GFP-Cas9, the CRISPR RNA (crRNA) and the trans-activating RNA (tracrRNA). Briefly, the crRNA associates with Cas9. This complex locates and binds the target DNA via base pairing interaction with the tracrRNA. The clones generated using this system did not produce targeted gene cleavage in Vero cells, CRISPRs (Sigma) were utilized in further gene editing experiments. The CRISPRs (Sigma) system utilizes a single plasmid that expresses both green fluorescent protein (GFP) and guide RNA (gRNA). Four guide strands were evaluated per gene target. Currently, we have successfully generated a KO cell line for the NEU2 gene in Vero cells. Other KO was attempted, and generation of other KO cell lines is on-going. The CRISPR (Sigma) sequences utilized in these experiments are presented in Table 3.

Gene editing was applied to generate KO vaccine cell lines (Sigma) using CRISPR-Cas selected genes was done in Vero cells maintained in Dulbecco’s modified Earle’s medium (DMEM, Invitrogen) supplemented with 10% fetal bovine serum. Lipofectamine LTX Reagent (Life Technologies) was utilized to transfect CRISPR plasmids into the Vero cells in 12-well culture plates, with a ratio of DNA/Lipofectamine at 1.0 ug/3.0 ul. Briefly, both 1.0 ug of CRISPR plasmid DNA and Lipofectamine were diluted into 100 ul of OPTI-MEM separately (Gibco Life Technologies; Carlsbad, CA). Diluted CRISPR plasmids were then mixed with transfection reagents and incubated at room temperature for 30 min. The cells were washed using ×1 PBS before the CRISPR/Lipofectamine mixture was added to the plate. Positive cells are sorted by flow cytometry into 96-well cell culture plates, with one cell/per well. Afterwards, the cell clones were grown in 12 well plates until confluence, and then divided into two plates: one plate was harvested for isolation of genomic DNA and then subjected to in-house Sanger sequencing; the second plate was frozen down at −80C until knockouts were validated by the sequencing data.

### Data analysis

For all experiments a non-targeting control siRNA (GE; D-001810-0X) was used as a negative control. The raw data was normalized to the non-targeting control results for the respective plate and then converted to a standard Z-score. For the initial screen hits were normalized and scored based on wells showing ≥3 s.d. above or below a non-targeting control. siRNAs that elicited an enhancement of viral replication>3 s.d.s. (z-score≥3.0) as compared to non-targeting control were considered top hits. These 76 genes were subjected to re-screening in Vero cells using SMARTpools.

## Data Records

### Data record 1

In this primary RNAi screen using small interfering RNA (siRNA), a library of siRNAs targeting human genes (GE Healthcare Dharmacon, # G-005005-01) was screened and a subset of genes in MA104 cells were identified that when silenced dramatically increased the ability of rotavirus replication. Also, included within this dataset, are the genes that when silenced dramatically decreased the ability of rotavirus replication. Sub-libraries included within this library: GPCR (G-103605-01), Phosphatase (G-103705-01), Ion Channel (G-103805-01), Drug targets (G-104655-025), Protease (G-105105-01), Ubiquitin (GU-105615-01, G-105625-01 and G-105635-01 for Ubiquitin Subset 1, 2 and 3 respectively), Protein Kinase (G-103505-01), and Genome (G-105005-025). Included within this dataset is the raw ELISA data and calculated z-scores for each gene hit. Anti-RV hits with a z-score greater than 1 are considered to be plausible antiviral host genes. The siRNA primary screen data presented is stored at PubChem (Data Citation 1).

### Data record 2

The top anti-RV hits from the primary screening assays were re-screened in a WHO-derived Vero vaccine cell line, which was the cell substrate to be used for enhanced vaccine cell line develop, to eliminate false-positive hits. In this case, individual siRNAs included in SMARTpools were tested to determine if they induced the phenotype observed of the pool, i.e., increased RV replication and changes in viral antigen production. Included within this dataset is the raw ELISA data for and calculated fold change for the host virus-resistant genes identified. Included within this dataset the host virus-resistant genes identified. This deconvolution validation dataset is stored at PubChem (Data Citation 2).

## Technical Validation

### Optimization of transfection in Vero cell line

**Figure 7:**
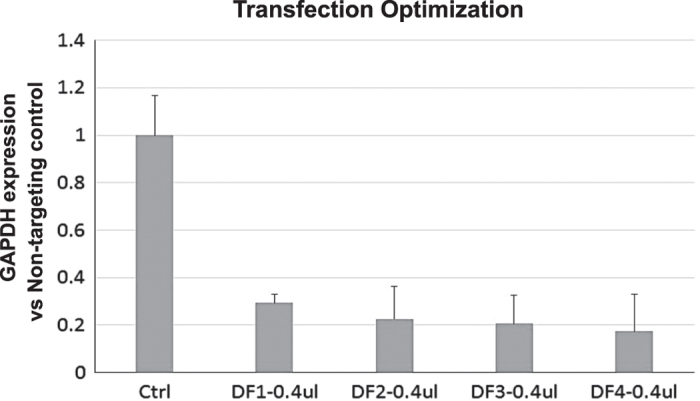
DharmaFECT 4 was the most effective transfection reagent for introducing siRNAs into MA104 cells. Prior to screening, DharmaFECT 1–4 were evaluated for their efficiency in delivering siRNAs to MA104 cells. A positive control siRNA targeting RV was transfected into MA104 cells using either 0.4 ul of DhamrmaFECT 1, 2, 3 or 4 following the protocol (ref). GAPDH expression was measured using QPCR assay. Results are presented as GAPDH expression versus non-targeting control.

**Figure 8:**
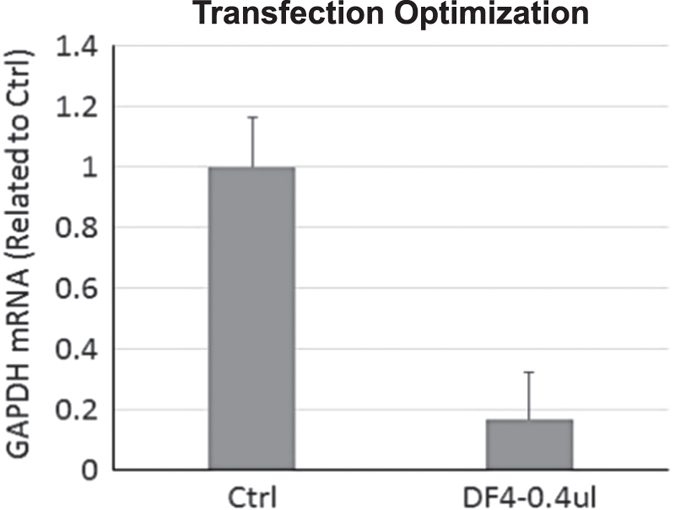
Transfection optimization. MA104 cells were transfected with DF4 and 50 nM GAPDH siRNA and allowed to incubate for 48 h. Following incubation cells were harvested to assess GAPDH silencing by qPCR. Shown is fold change in GAPDH mRNA following transfection with anti-GAPDH siRNA relative to control with no transfection reagent. DF4 at 0.4% concentration showed the highest mRNA reduction with the lowest toxicity as compared to DF1, DF2 and DF3.

To establish a highly efficient siRNA transfection protocol for high-throughput screening (HTS), the effectiveness of four DharmaFECT transfection reagents (DF1–4, Dharmacon) were tested with MA104 cells. From this collection, DF4 was identified to be the most effective reagent for introducing siRNAs into the cell line for primary screening (Fig. 7). A result showing optimal DF4 transfection conditions is shown in Fig. 8. Using a siRNA targeting the GAPDH gene, >80% silencing was achieved with 0.4% DF4 and a siRNA concentration of 50 nM. These conditions are consistent with previously published data^12^. Under these conditions, cell toxicity was not observed by either microscopy examination or MTT assay. Transfection optimization for the Vero cell line (used for validation studies) was previously performed during the polio cell line engineering program^12^. Pilot studies were completed prior to screening to develop an assay with low variation and strong signal to noise ratio. Effective concentrations of siRNAs were determined by titration. Responses to negative and positive control siRNAs were evaluated and optimized to determine reference values and the s.d. for the z-score calculation. Z-scores were subsequently used to evaluate and determine hits.

### Transfection controls

All experiments were performed in at least two independent replicates. Each replicate included duplicates for each siRNA targeting a specific mRNA transcript for the gene of interest, and quadruplicates of a positive control siRNA targeting Rotavirus, a negative non-targeting control siRNA and a transfection control siRNA (siTOX). A high-quality RNAi screen requires a clear distinction between positive and negative controls. A control siRNA that targets Rotavirus directly was utilized to monitor the silencing effects on rotavirus replication (NSP-842: AGTTGAACGTGACGACAATT). A non-targeting siRNA (siNTC) (GE Healthcare Dharmacon: Cat. # D-001810-0X) was used as a negative control. These controls were clearly distinguished from each other in all experiments. A mock control (no siRNA) was used as background normalization. The effectiveness of these controls is outline in Fig. 7. In an effort to evaluate screening efficiency, we used siTOX (GE Healthcare Dharmacon TOX Transfection Control, Cat. # D0015000120) to visually monitor the transfection efficiency. Successful transfection with siTOX results in cell death by apoptosis.

### Toxicity and cellular viability screening

All siRNAs were tested for effects on cellular viability and toxicity by CellTiter96 Non-Radioactive Cell Proliferation Assay Kit and ToxiLight bioassay (Lonza, Walkersville, MD) following manufacturer’s instructions. Cellular toxicity was evaluated 48 h post-transfection. Data was normalized to siTOX control (100% cytotoxicity). siRNAs which resulted in >20% toxicity were excluded as is standard operating procedure.

### Data normalization and statistical analysis

In the current study, we used Z-score analysis according to previously established methods^13–15^. A Z′-factor was utilized in the current screen (3>Z′>0.5: Excellent assay; 0.5>Z′>3: acceptable) to evaluate its quality. The majority of our screen fell into a Z′ factor (0.5<Z′<3.0) that reflects a high-quality primary screen. The primary screen data was normalized to the whole 96-well plate to set the mean (μ) of the data to zero and the s.d. to 1. The raw ELISA absorbance data values were normalized. The mean of the replicates where calculated and standardized using Z-score analysis. The mean values of the experimental siRNA treated wells were compared to the mean of the negative control treated wells. Z-score was calculated by determining the difference between the normalized score and the mean of the plate. This value was then divided by the s.d. of the plate. Positive hits from the primary screen are scored by Z-score≥3 s.d. Statistical analysis was completed by using a one-way ANOVA with Turkey’s multiple comparisons at 95% confidence level. All data are presented as mean±s.e. *P*-values <0.05 were considered statistically significant.

## Additional Information

**How to cite this article:** Wu, W. *et al.* Development of improved vaccine cell lines against rotavirus. *Sci. Data* 4:170021 doi: 10.1038/sdata.2017.21 (2017).

**Publisher’s note:** Springer Nature remains neutral with regard to jurisdictional claims in published maps and institutional affiliations.

## Supplementary Material



## Figures and Tables

**Table 1:** Top 10 gene hits in Vero cells following deconvolution and subsequent validation.

**Gene ID**	**Official Gene Name**	**Entrez Number**	**SMARTpool Catalog Number**
NEU2	Neuraminidase 2 (Cytosolic sialidase)	4,759	L-012058-02-0005
NAT9	N-acetyltransferase 9 (Putative)	26,151	L-017635-02-0005
COQ9	Coenzyme Q9	57,017	L-022565-02-0005
SVOPL	Synaptic vesicle glycoprotein 2 (SV2) related protein homolog	1,36,306	L-007346-01-0005
NDUFA9	NADH:ubiquinone oxidoreductase subunit A9	4,704	L-016044-01-0005
COX20	Cytochrome c oxidase assembly factor	1,16,228	L-021429-01-0005
LRGUK	Leucine-rich repeats and guanylate kinase containing domain	1,36,332	L-015997-01-0005
WDR62	WD repeat domain 62	2,84,403	L-015997-01-0005
RAD51AP1	RAD51 recombinase associated protein 1	10,635	L-017166-00-0005
CDK6	Cyclin-dependent kinase 6	1,021	L-003240-00-0005

**Table 2:** The top 76 hits were re-screened in a Vero vaccine cell line utilizing SMARTpools.

**Gene Target**	**Entrez ID**	**Deconvoluted siRNA**	**Catalog number**	**Fold changes (versus NTC)**
None		NTC	D-001810-0X	1
NEU2	4,759	siRNA 1	J-012058-17	1.196
NEU2	4,759	sIRNA 2	J-012058-18	1.465
NEU2	4,759	siRNA 3	J-012058-19	1.353
NEU2	4,759	siRNA 4	J-012058-20	1.606
NAT9	26,151	siRNA 1	J-017635-13	0.823
NAT9	26,151	sIRNA 2	J-017635-14	1.241
NAT9	26,151	siRNA 3	J-017635-15	1.484
NAT9	26,151	siRNA 4	J-017635-16	1.442
SVOPL	1,36,306	siRNA 1	J-007346-13	0.777
SVOPL	1,36,306	sIRNA 2	J-007346-14	1.48
SVOPL	1,36,306	siRNA 3	J-007346-15	1.632
SVOPL	1,36,306	siRNA 4	J-007346-16	1.044
COQ9	57,017	siRNA 1	J-022565-18	0.948
COQ9	57,017	sIRNA 2	J-022565-19	1.531
COQ9	57,017	siRNA 3	J-022565-20	1.477
COQ9	57,017	siRNA 4	J-022565-21	1.26
NDUFA9	4,704	siRNA 1	J-016044-09	1.085
NDUFA9	4,704	sIRNA 2	J-016044-10	1.648
NDUFA9	4,704	siRNA 3	J-016044-11	1.641
NDUFA9	4,704	siRNA 4	J-016044-12	1.233
RAD51AP1	10,635	siRNA 1	J-017166-05	1.094
RAD51AP1	10,635	sIRNA 2	J-017166-06	1.217
RAD51AP1	10,635	siRNA 3	J-017166-07	1.568
RAD51AP1	10,635	siRNA 4	J-017166-08	1.478
COX20	1,16,228	siRNA 1	J-021429-09	1.021
COX20	1,16,228	sIRNA 2	J-021429-10	1.478
COX20	1,16,228	siRNA 3	J-021429-11	1.602
COX20	1,16,228	siRNA 4	J-021429-12	0.937
MAPK6	5,597	siRNA 1	J-003594-11	1.361
MAPK6	5,597	sIRNA 2	J-003594-12	1.405
MAPK6	5,597	siRNA 3	J-003594-13	1.378
MAPK6	5,597	siRNA 4	J-003594-14	1.067
WDR62	2,84,403	siRNA 1	J-031771-09	1.361
WDR62	2,84,403	sIRNA 2	J-031771-10	1.485
WDR62	2,84,403	siRNA 3	J-031771-11	1.402
WDR62	2,84,403	siRNA 4	J-031771-12	1.622
LRGUK	1,36,332	siRNA 1	J-015997-09	1.101
LRGUK	1,36,332	sIRNA 2	J-015997-10	1.585
LRGUK	1,36,332	siRNA 3	J-015997-11	1.161
LRGUK	1,36,332	siRNA 4	J-015997-12	1.568
CDK6	1,021	siRNA 1	J-003240-10	1.315
CDK6	1,021	siRNA 2	J-003240-11	1.434
CDK6	1,021	siRNA 3	J-003240-12	1.529
CDK6	1,021	siRNA 4	J-003240-13	0.951
KIAA1683	80,726	siRNA 1	J-031251-05	1.158
KIAA1683	80,726	siRNA 2	J-031251-06	1.242
KIAA1683	80,726	siRNA 3	J-031251-07	1.24
KIAA1683	80,726	siRNA 4	J-031251-08	1.097
CRISP3	10,321	siRNA 1	J-020092-09	0.969
CRISP3	10,321	siRNA 2	J-020092-10	1.454
CRISP3	10,321	siRNA 3	J-020092-11	1.436
CRISP3	10,321	siRNA 4	J-020092-12	1.218
GRPR	2,925	siRNA 1	J-005624-05	1.039
GRPR	2,925	siRNA 2	J-005624-06	1.535
GRPR	2,925	siRNA 3	J-005624-07	1.925
GRPR	2,925	siRNA 4	J-005624-08	0.971
DPH7	92,715	siRNA 1	J-016561-09	1.121
DPH7	92,715	siRNA 2	J-016561-10	1.343
DPH7	92,715	siRNA 3	J-016561-11	1.573
DPH7	92,715	siRNA 4	J-016561-12	1.275
GEMIN8	54,960	siRNA 1	J-020841-17	1.1
GEMIN8	54,960	siRNA 2	J-020841-18	1.047
GEMIN8	54,960	siRNA 3	J-020841-19	1.501
GEMIN8	54,960	siRNA 4	J-020841-20	1.437
KIAA1407	57,577	siRNA 1	J-014068-17	1.154
KIAA1407	57,577	siRNA 2	J-014068-18	1.292
KIAA1407	57,577	siRNA 3	J-014068-19	1.424
KIAA1407	57,577	siRNA 4	J-014068-20	1.089
RFXAP	5,994	siRNA 1	J-011104-05	1.005
RFXAP	5,994	siRNA 2	J-011104-06	1.514
RFXAP	5,994	siRNA 3	J-011104-07	1.532
RFXAP	5,994	siRNA 4	J-011104-08	1.669
SMARCA4	6,597	siRNA 1	J-010431-05	1.493
SMARCA4	6,597	siRNA 2	J-010431-06	1.51
SMARCA4	6,597	siRNA 3	J-010431-07	1.462
SMARCA4	6,597	siRNA 4	J-010431-08	1.698
CFAP58	1,59,686	siRNA 1	J-026312-17	1.452
CFAP58	1,59,686	siRNA 2	J-026312-18	1.4
CFAP58	1,59,686	siRNA 3	J-026312-19	1.366
CFAP58	1,59,686	siRNA 4	J-026312-20	1.558
The top 20 hits that repeated the primary screen’s findings were subjected to deconvolution studies. The data for these hits is outlined here. Data is presented as fold change compared to non-targeting siRNA control.				

**Table 3:** Knockouts of the top 10 hits identified by the primary and validation RNAi screens were generated in Vero cells using CRISPR-Cas9.

**Gene symbol**	**Gene ID**	**RefSeq ID**	**Species**	**Target exon**	**Order format**	**Target site**
NEU2	4,759	NM_005383	Human	1	Plasmid(U6-gRNA/CMV-Cas9-GFP)	AGGAGAGCGTGTTCCAGTCGGG
NEU2	4,759	NM_005383	Human	1	Plasmid(U6-gRNA/CMV-Cas9-GFP)	TGTTCCGCGAAGGCCAGCAGGG
NEU2	4,759	NM_005383	Human	1	Plasmid(U6-gRNA/CMV-Cas9-GFP)	GCTGGCCTTCGCGGAACAGCGG
NEU2	4,759	NM_005383	Human	1	Plasmid(U6-gRNA/CMV-Cas9-GFP)	AGCTGATTGTCCTGCGCAGAGG
NAT9	26,151	NM_015654	Human	2	Plasmid(U6-gRNA/CMV-Cas9-GFP)	CTCCGAGGTGTAGGGTACAAGG
NAT9	26,151	NM_015654	Human	3	Plasmid(U6-gRNA/CMV-Cas9-GFP)	CTCTGATTTCATCCACTCGTGG
NAT9	26,151	NM_015654	Human	4	Plasmid(U6-gRNA/CMV-Cas9-GFP)	TCTTCGGTGGCGCCTGGCTGGG
NAT9	26,151	NM_015654	Human	4	Plasmid(U6-gRNA/CMV-Cas9-GFP)	AGCTCTCTTCGGTGGCGCCTGG
SVOPL	1,36,306	NM_001139456	Human	1	Plasmid(U6-gRNA/CMV-Cas9-GFP)	CGGCGGTATCTGGTGCGCTTGG
SVOPL	1,36,306	NM_001139456	Human	1	Plasmid(U6-gRNA/CMV-Cas9-GFP)	GGTATCTGGTGCGCTTGGCCGG
SVOPL	1,36,306	NM_001139456	Human	2	Plasmid(U6-gRNA/CMV-Cas9-GFP)	ACGCGGCACCAGGGCTTGTCGG
SVOPL	1,36,306	NM_001139456	Human	2	Plasmid(U6-gRNA/CMV-Cas9-GFP)	AAACTCAAGGGCTGCCGTCAGG
COQ9	57,017	NM_020312	Human	1	Plasmid(U6-gRNA/CMV-Cas9-GFP)	GCTCAATTTCCGAAGGCTGAGG
COQ9	57,017	NM_020312	Human	1	Plasmid(U6-gRNA/CMV-Cas9-GFP)	GCCTTCGGAAATTGAGCCTGGG
COQ9	57,017	NM_020312	Human	2	Plasmid(U6-gRNA/CMV-Cas9-GFP)	CTTCCACGGTGAACGTCTTTGG
COQ9	57,017	NM_020312	Human	3	Plasmid(U6-gRNA/CMV-Cas9-GFP)	CAGTGGAGACTATCGGCTTCGG
NDUFA9	4,704	NM_005002	Human	4	Plasmid(U6-gRNA/CMV-Cas9-GFP)	GCTGTGTTGTACTACTCGTCGG
NDUFA9	4,704	NM_005002	Human	9	Plasmid(U6-gRNA/CMV-Cas9-GFP)	CGGTACCTCCTTTTCCACCTGG
NDUFA9	4,704	NM_005002	Human	9	Plasmid(U6-gRNA/CMV-Cas9-GFP)	CGATAGGCAAAAAGCGGCAAGG
NDUFA9	4,704	NM_005002	Human	11	Plasmid(U6-gRNA/CMV-Cas9-GFP)	GCGTCATCGCACTTACCGCTGG
The CRISPR (Sigma) sequences utilized in these experiments are presented (4 target sites per gene).						

## References

[d1] NCBI PubChem BioAssay Wu W. Orr-Burks N. Karpilow J. Tripp R. 2016 1224833

[d2] NCBI PubChem BioAssay Wu W. Orr-Burks N. Karpilow J. Tripp R. 2016 1224832

